# Cerebrospinal fluid glucose-to-lactate ratio (CGLR) as a diagnostic biomarker for postoperative intracranial infections in patients with acute brain injury: a prospective diagnostic accuracy study

**DOI:** 10.3389/fmed.2026.1830485

**Published:** 2026-06-10

**Authors:** Weidong Wang, Huajun Wang, Chengjie Zhou, Ye Fu

**Affiliations:** Department of Intensive Care Unit, The Affiliated People’s Hospital of Ningbo University, Ningbo, China

**Keywords:** acute brain injury, cerebrospinal fluid glucose, cerebrospinal fluid glucose-to-lactate, cerebrospinal fluid lactate, postoperative intracranial infection

## Abstract

**Objectives:**

The aim of this study was to evaluate the diagnostic accuracy of the cerebrospinal fluid glucose-to-lactate ratio (CGLR) for detecting postoperative intracranial infections in patients with acute brain injury (ABI) and compare it with conventional biomarkers.

**Methods:**

A single-center prospective study was conducted with 121 postoperative ABI patients suspected of infection. Cerebrospinal fluid (CSF) and peripheral blood samples were collected under aseptic conditions. Levels of CSF lactate (cLac), CSF glucose (cGlu), CSF procalcitonin (PCT), and CGLR were measured.

**Results:**

The AUC values for cGlu and cLac were 0.836 and 0.820, respectively, with optimal cutoffs of 2.6 mmol⋅L^–1^ for cGlu and 4.5 mmol⋅L^–1^ for cLac. The combined metric, CGLR, demonstrated a higher diagnostic performance (AUC = 0.866) with an optimal cutoff of 0.61. CGLR values were similar between hyperglycemic and normoglycemic patients, regardless of infection status (all *P* > 0.05). The diagnostic performance of CGLR was consistent across SAH, ICH, and TBI patient cohorts (all *P* > 0.05).

**Conclusion:**

CGLR is a rapid, cost-effective, and reliable biomarker for diagnosing postoperative intracranial infections in ABI patients. It outperforms traditional markers and remains unaffected by peripheral glucose fluctuations.

## 1 Introduction

Acute brain injury (ABI), encompassing subarachnoid hemorrhage (SAH), intracranial hemorrhage (ICH), and traumatic brain injury (TBI), is characterized by high mortality and disability rates. ABI represents a frequently encountered severe condition in neurosurgery resulting from head trauma (1). Surgical intervention is often lifesaving (2). However, postoperative intracranial infection remains a major challenge in ABI management and a critical risk factor for mortality (3–5). Early detection of intracranial infection is essential. Although cerebrospinal fluid (CSF) bacterial culture is the standard criterion for diagnosing postoperative infection, it has limitations, including susceptibility to contamination, prolonged processing times, and low positivity rates (6). Thus, identifying CSF biomarkers that reliably predict postoperative intracranial infection could enable timely treatment.

CSF lactate (cLac), CSF glucose (cGlu), and CSF procalcitonin (cPCT) have demonstrated usefulness in diagnosing postoperative intracranial infection (7–10). Determining cLac levels is cost-effective, easily accessible, and unaffected by leukocyte or erythrocyte presence in CSF (8). Although the CSF glucose-to-lactate ratio (CGLR) has recently been associated with ABI prognosis, its relationship with postoperative intracranial infection remains unclear (11, 12).

This study aims to evaluate the reliability of CGLR as a biomarker for postoperative intracranial infection following ABI and compare its diagnostic performance with traditional markers (cLac, cGlu, and CSF PCT).

## 2 Materials and methods

### 2.1 Study design and participants

This prospective observational study was conducted at a single center (Intensive Care Unit, People’s Hospital of Ningbo University) from June 2024 to June 2025. Patients with ABI who had undergone surgery and subsequently exhibited fever and suspicion of intracranial infection were included. CSF samples (obtained via lumbar puncture) and serum samples were collected within 24 h. Exclusion criteria included: (1) diagnosis of sepsis, (2) history of other neurosurgical procedures, (3) contraindications to lumbar puncture, (4) renal failure, (5) age under 18 years, (6) pregnancy, (7) hypoxic-ischemic encephalopathy, and (8) epilepsy. Each sample was collected from a unique patient, with no repeated sampling from the same individual. The study complied with the Declaration of Helsinki (2000) and was approved by the Ethics Committee of the Affiliated People’s Hospital of Ningbo University (2024058).

### 2.2 Diagnostic criteria

Diagnostic criteria for intracranial infection were based on the CDC/NHSN Surveillance Definitions (13). Patients were diagnosed with intracranial infection if they met either of the following conditions: (1) identification of a pathogen through positive CSF culture or confirmation via next-generation sequencing (NGS); or (2) presence of at least one clinical symptom not attributable to other causes (e.g., temperature above 38°C, meningeal irritation signs, focal neurological deficits, or headache) along with at least one of the following: positive blood culture, positive CSF Gram staining, or abnormal CSF parameters (white blood cell count > 100 cells/mm^3^, protein > 50 mg/dL, and glucose < 2.5 mmol/L).

In this study, CSF underwent next-generation sequencing (NGS) with approval from patients or their families.

### 2.3 Sample collection

Causes of ABI, including TBI, ICH, and SAH, were recorded. Basic demographic data (age, gender) and clinical findings were also collected. Levels of leukocytes, lactate, glucose, and PCT were measured in serum and CSF for both groups.

CSF samples were collected from all participants by lumbar puncture. The GEM Premier 4,000 system (United States) was used as an arterial blood gas analyzer to measure lactate and glucose levels. The enzyme-linked fluorescence method (Marcy L’Etoile, France) was used to determine PCT concentrations, with a minimum detection threshold of 0.05 ng/mL. Under sterile conditions, peripheral venous blood samples were collected within 30 min after obtaining CSF.

All CSF samples were analyzed within 30 min of collection and stored at 4°C until analysis. No freeze-thaw cycles occurred prior to glucose and lactate measurements.

### 2.4 Statistical analysis

Based on a preliminary CGLR AUC of 0.85 from a pilot study of 20 patients, a minimum sample size of 90 patients was required (power of 0.8, alpha of 0.05). To account for potential exclusions, 121 patients were enrolled.

Statistical analysis was performed using SPSS software (version 24.0, IBM Corporation, Armonk, NY, United States). Continuous variables with normal distribution are expressed as mean ± standard deviation (SD), while skewed data are presented as median (interquartile range, IQR). Categorical variables are presented as numbers (percentages). Differences between groups were assessed using the Chi-square test for categorical variables. The Mann-Whitney U test or Student’s *t*-test were used for continuous variables. ROC curves were constructed to evaluate diagnostic performance. *P* < 0.05 were considered statistically significant. The statistical analysis plan was finalized prior to data collection and remained unchanged after data access.

## 3 Results

### 3.1 Study population

**FIGURE 1:**
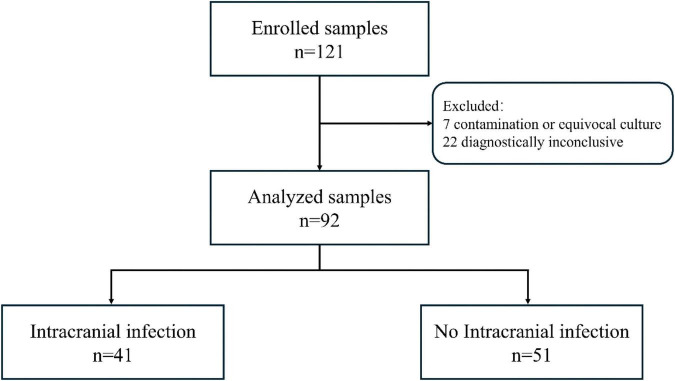
Flow diagram of the study. Among the 121 initial CSF samples, 7 were excluded due to likely contamination or equivocal culture results (defined as growth of skin commensals such as coagulase-negative staphylococci, with CSF parameters showing white blood cell count < 100 cells/mmł, protein < 50 mg/dL, and glucose ≥ 2.5 mmol/L, indicating no definitive evidence of intracranial infection). An additional 22 samples were classified as diagnostically inconclusive (defined as ambiguous clinical or microbiological findings that precluded a definitive diagnosis of intracranial infection, e.g., borderline CSF leukocyte count 100–300 cells/mm^3^ without positive culture or NGS). The remaining 92 samples, which met clear criteria for either intracranial infection (*n* = 41) or no infection (*n* = 51), were included in the final analysis.

A total of 121 CSF samples were collected during the study period. Of these initial samples, 7 were excluded due to contamination or equivocal culture results. Another 22 samples were classified as diagnostically inconclusive. The remaining 92 samples, clearly defined as either intracranial infection (*n* = 41) or no infection (*n* = 51), were included in the final analysis (Figure 1). Clinical characteristics of enrolled participants are presented in Table 1.

**TABLE 1:** Characteristics of samples stratified according to confirmed intracranial infection and no intracranial infection groups.

Characteristics	Intracranial infection (*n* = 41)	No Intracranial infection (*n* = 51)	*P*-value
Ages (year)	59.5 ± 13.30	57.8 ± 15.56	0.584
Sex (male)	26 (63.41%)	34 (66.67%)	0.745
Type of diseases			
TBI	13 (31.71%)	17 (33.33%)	0.585
ICH	21 (51.22%)	26 (50.98%)	0.560
SAH	7 (17.07%)	8 (15.69%)	1.000
Clinical findings			
Diabetes mellitus	10 (24.39%)	21 (41.18%)	0.088
On dexamethasone	4 (9.76%)	7 (13.73%)	0.565
GCS	6.10 ± 1.59	6.61 ± 1.56	0.126
Blood parameters			
WCC (×10^9^/L)	10.36 ± 4.80	11.35 ± 3.90	0.367
PCT (ng/ml) *	0.15 (0.10–0.29)	0.15 (0.08–0.42)	0.778*
bGlu (mmol/L) *	8.40 (7.40–11.10)	9.90 (6.80–13.7)	0.646*
bLac (mmol/L) *	1.40 (1.00–1.85)	1.50 (1.10–2.10)	0.431*
CSF parameters			
cLac (mmol/L) *	5.80 (4.10–7.75)	3.20 (2.20–4.30)	< 0.001*
cGlu (mmol/L) *	2.10 (1.65–3.30)	4.40 (3.30–5.50)	< 0.001*
cPro (mg/L) *	2504.00 (1196.50–3387.50)	946.75 (535.50–2220.25)	0.001*
CSF leukocyte (/uL) *	875.00 (150.00–2868.00)	55.00 (5.00–467.50)	< 0.001*
CSF PCT (ng/ml) *	0.17 (0.12–0.28)	0.13 (0.095–0.21)	0.030*
CGLR*	0.38 (0.24–0.60)	1.45 (0.81–2.12)	< 0.001*

TBI, traumatic brain injury; ICH, intracranial hemorrhage; SAH, subarachnoid hemorrhage; GCS, Glasgow Coma Scale; WCC, white cell count; PCT, procalcitonin; bGlu, blood glucose level; bLac, blood lactate level; CSF, cerebrospinal fluid; cLac, cerebrospinal fluid lactate; cGlu, cerebrospinal fluid glucose; cPro, cerebrospinal fluid protein; CGLR, cerebrospinal fluid glucose-to-lactate ratio; IQR, interquartile range; SD, standard deviation. *Data presented in median (IQR). The rest of data presented in n (%) or mean SD.

Microbiological findings are summarized in Table 2. Among the 41 infected patients, 17 (41.5%) had positive CSF culture or NGS results. Monomicrobial infections were identified in 10 cases (58.8%), and polymicrobial infections in 7 cases (41.2%).

**TABLE 2:** Microbiological findings in patients with postoperative intracranial infection (*n* = 41).

Category	*n*	Proportion (%)
Total infected patients	41	100
Culture/NGS positive	17	41.5
Culture/NGS negative	24	58.5
Among culture/NGS positive (n = 17)		
Monomicrobial infections	10	58.8
Polymicrobial infections	7	41.2
Monomicrobial infections (n = 10)		
Acinetobacter baumannii	4	40.0
Stenotrophomonas maltophilia	2	20.0
Roseomonas mucosa	1	10.0
Staphylococcus haemolyticus	1	10.0
Staphylococcus caprae	1	10.0
Chlamydia pneumonia	1	10.0
Polymicrobial infections (n = 7)		
Enterococcus faecium + Staphylococcus hominis	1	14.3
Acinetobacter baumannii + Aspergillus fumigatus	1	14.3
Porphyromonas gingivalis + Parvimonas micra + Fusobacterium nucleatum	1	14.3
Pseudomonas aeruginosa + Acinetobacter baumannii	1	14.3
Klebsiella pneumoniae + Cytomegalovirus	1	14.3
Streptococcus pneumoniae + Klebsiella pneumoniae	1	14.3
Corynebacterium accolens + Candida parapsilosis	1	14.3

**FIGURE 2:**
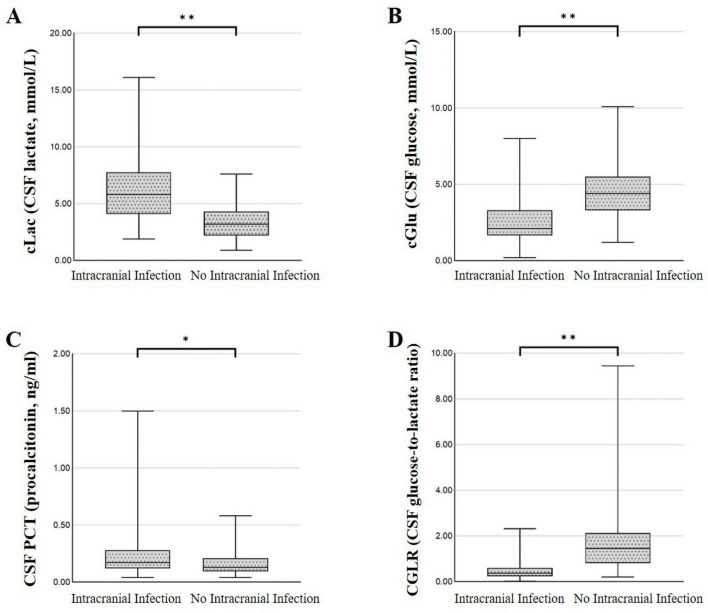
Comparison of cerebrospinal fluid lactate level **(A)**, glucose level **(B)**, procalcitonin level **(C)** and cerebrospinal fluid glucose-to-lactate ratio **(D)** between intracranial infection groups and no intracranial infection groups, presented in median and interquartile range. cLac, cerebrospinal fluid lactate; CSF, cerebrospinal fluid; cGlu, cerebrospinal fluid glucose; PCT, procalcitonin; CGLR, cerebrospinal fluid glucose-to-lactate ratio. **P < 0.001 (Mann-Whitney U test); *P < 0.05 (Mann-Whitney U test).

No significant differences existed between infected and non-infected groups regarding age, gender, disease type, clinical findings, or blood parameters (*P* > 0.05). However, significant differences emerged in CSF parameters. Levels of cPro, cLac, and CSF leukocytes were significantly elevated in the intracranial infection group (*P* < 0.005). Additionally, CSF PCT concentrations were higher in infected patients (*P* < 0.05). The intracranial infection group also showed significantly reduced CGLR and cGlu (*P* < 0.001) (Figure 2 and Table 1).

### 3.2 Comparison of systemic (bGlu, bLac) and central (cGlu, cLac, CGLR) biomarkers

**FIGURE 3:**
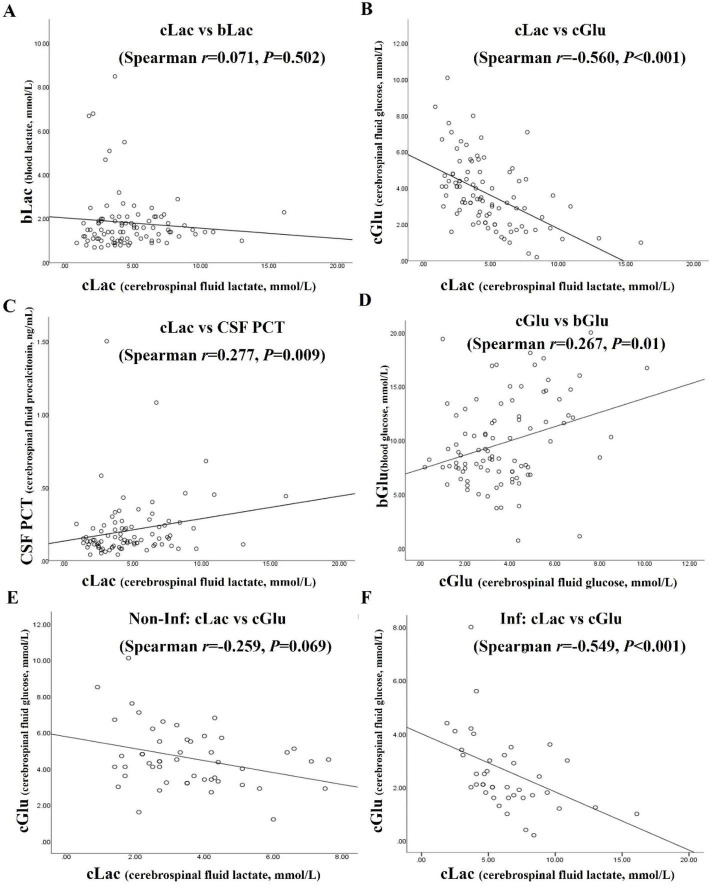
Associations involving cerebrospinal fluid lactate and blood lactate appear in **(A)**, with cerebrospinal fluid glucose in panel **(B)**, plus cerebrospinal fluid procalcitonin in **(C)**. **(D)** The relationship linking cerebrospinal fluid glucose to blood glucose. Such linkage emerges for the group lacking intracranial infection within **(E)**. **(F)** This connection amid the intracranial infection cohort. cLac denotes cerebrospinal fluid lactate; bLac, blood lactate; cGlu, cerebrospinal fluid glucose; CSF, cerebrospinal fluid; PCT, procalcitonin; bGlu, blood glucose; Inf, intracranial infection; Non-Inf, no intracranial infection.

A weak but significant correlation existed between glucose levels in CSF and blood (*r* = 0.267, *P* = 0.01), while no significant association was observed for lactate (*r* = 0.071, *P* = 0.502) (Figures 3A–D). Meanwhile, cLac levels showed a moderate negative correlation with cGlu (Spearman *r* = -0.560, *P* < 0.001), whereas a minimal association appeared between CSF PCT and cLac (Figures 3B,C).

Subgroup analysis revealed a weak negative correlation without statistical significance between lactate and CSF glucose in patients without infection (*r* = **-**0.259, *P* = 0.069) (Figure 3E). In contrast, patients with infection showed a strong, significant negative correlation for these parameters (*r* = **-**0.549, *P* < 0.001) (Figure 3F).

**FIGURE 4:**
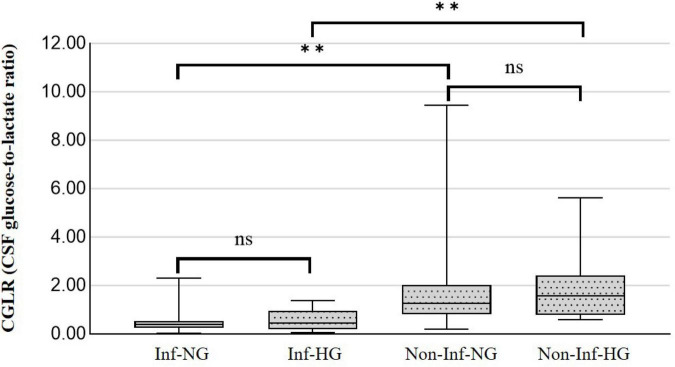
Comparison of CGLR among patient subgroups stratified by infection and glycemic status. Inf-NG, intracranial infection with normoglycemia; Inf-HG, intracranial infection with hyperglycemia; Non-Inf-NG, no intracranial infection with normoglycemia; Non-Inf-HG, no intracranial infection with hyperglycemia. **P < 0.001 (Mann-Whitney U test).

Patients were stratified into normoglycemic and hyperglycemic subgroups using a blood glucose cutoff of 11.1 mmol/L (14). No significant differences appeared in CGLR between normoglycemic and hyperglycemic subgroups, regardless of intracranial infection (both *P* > 0.05). However, CGLR was significantly lower in infected patients compared to non-infected patients within each subgroup (both *P* < 0.001) (Figure 4).

**FIGURE 5:**
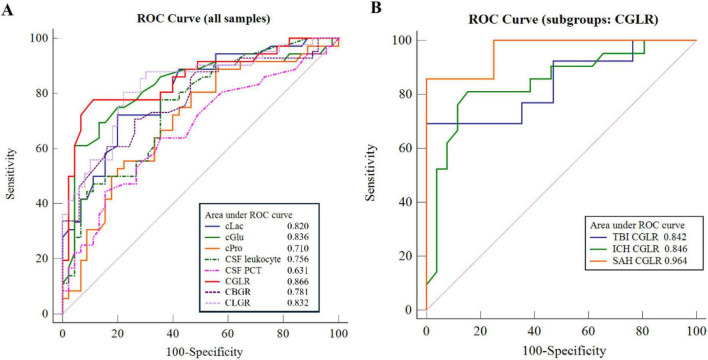
ROC curve analysis of cLac, cGlu, c*P*ro, CSF leukocyte, CSF PCT, and CGLR presented **(A)**. ROC analysis: comparing the efficacy of CGLR in diagnosing intracranial infection across different acute brain injury subgroups **(B)**. cLac, cerebrospinal fluid lactate; cGlu, cerebrospinal fluid glucose; cPro, cerebrospinal fluid protein; CSF, cerebrospinal fluid; PCT, procalcitonin; CGLR, cerebrospinal fluid glucose-to-lactate ratio; CBGR, cerebrospinal fluid glucose-to-blood glucose ratio; CLGR, cerebrospinal fluid lactate-to-CBGR ratio; TBI, traumatic brain injury; ICH, intracranial hemorrhage; SAH, subarachnoid hemorrhage.

ROC analysis showed an AUC of 0.836 for cGlu with an optimal cutoff of 2.6 mmol/L (Figure 5A and Table 3). cLac had an AUC of 0.820 at a cutoff of 4.5 mmol/L. The CSF-to-blood glucose ratio (CBGR) showed moderate diagnostic accuracy (AUC 0.781, Youden index 0.45), whereas the combined lactate-to-glucose ratio (CLGR) demonstrated good accuracy (AUC 0.832, Youden index 0.58). CGLR had the best diagnostic performance, with the highest AUC (0.866), specificity (90.0%), and Youden index (0.68) among all biomarkers.

**TABLE 3:** ROC curve analysis results of CSF marker in differentiating intracranial infection and no intracranial infection.

CSF Maikers	AUC(95% CI)	Cut-off point	Sensitivity, %	Specificity, %	PPV, %	NPV, %	Youden index
cLac (mmol/L)	0.820 (0.726–0.893)	>4.5	73.17	82.00	76.9	78.8	0.55
cGlu (mmol/L)	0.836 (0.744–0.905)	≤ 2.6	63.41	96.00	92.9	76.2	0.59
cPro (mg/L)	0.710 (0.600–0.804)	>1,079	80.56	55.32	58.0	78.8	0.36
CSF leukocyte (/uL)	0.756 (0.652–0.843)	>110	78.38	63.27	61.7	79.5	0.42
CSF PCT (ng/ml)	0.631 (0.521–0.732)	>0.22	41.03	83.33	66.7	63.5	0.24
CBGR	0.781 (0.683–0.861)	≤ 0.29	60.98	84.00	75.8	72.4	0.45
CLGR	0.832 (0.740–0.903)	>12.32	80.49	78.00	75.0	83.0	0.58
CGLR	0.866 (0.779–0.929)	≤ 0.61	78.05	90.00	86.5	83.3	0.68

cLac, cerebrospinal fluid lactate; cGlu, cerebrospinal fluid glucose; cPro, cerebrospinal fluid protein; CSF, cerebrospinal fluid; PCT, procalcitonin; CBGR, cerebrospinal fluid glucose-to-blood glucose ratio; CLGR, cerebrospinal fluid lactate-to-CBGR ratio; CGLR, cerebrospinal fluid glucose-to-lactate ratio.

Additional subgroup analyses were performed. CGLR exhibited superior diagnostic accuracy in patients with SAH, ICH, and TBI, with corresponding AUC values of 0.964, 0.846, and 0.842, respectively (Figure 5B). Differences among these groups were not significant (all *P* > 0.05) (Table 4). Optimal cutoff values for each subgroup, determined by the Bootstrap method, were 0.53, 0.61, and 0.51, respectively. The 95% confidence intervals for these values extensively overlapped (0.38–1.14, 0.59–1.37, 0.30–0.92) (Table 4).

**TABLE 4:** Diagnostic performance of CGLR for intracranial infection: comparison across etiology subgroups.

Groups	AUC (95% CI)	Cut-off point	Bootstrap 95% CI for Cut-off	Sensitivity, %	Specificity, %	*P*-value (vs. TBI)	*P*-value (vs. ICH)	*P*-value (vs. SAH)	PPV, %	NPV, %	Youden index
TBI (*n* = 30)	0.842 (0.663–0.948)	≤ 0.53	≤0.38 to ≤ 1.14	69.23	100.00	–	0.9640	0.1740	100.0	81.0	0.69
ICH (*n* = 47)	0.846 (0.711–0.935)	≤ 0.61	≤0.59 to ≤ 1.37	80.95	84.62	0.9640	–	0.1123	81.0	84.6	0.66
SAH (*n* = 15)	0.964 (0.725–1.000)	≤ 0.51	≤0.3 to ≤ 0.92	85.71	100.00	0.1740	0.1123	–	100.0	88.9	0.86

CGLR, cerebrospinal fluid glucose-to-lactate ratio glucose-to-lactate ratio; TBI, traumatic brain injury; ICH, intracranial hemorrhage; SAH, subarachnoid hemorrhage.

### 3.3 Multivariable analyses

Independent predictors associated with intracranial infection were determined using multivariable logistic regression analysis. The model included variables significant in the univariate analysis (CGLR, CSF leukocyte, cPro, and CSF PCT) but excluded cGlu and cLac due to intrinsic collinearity with CGLR. In multivariate analysis, CGLR was the only independent predictor significantly associated with intracranial infection (OR: 0.112; 95% CI: 0.040–0.318; *P* < 0.001). In contrast, CSF PCT, CSF leukocyte count, and CSF protein did not retain independent significance.

## 4 Discussion

Glucose is a critical substrate for maintaining normal brain metabolism and function. In patients with postoperative intracranial infections after ABI, reduced brain glucose availability results from impaired cerebral blood flow, bacterial metabolism, neutrophil glycolysis, and anaerobic metabolism in brain tissue (7, 15). Recent studies have demonstrated rapid activation of microglial cells during inflammation or brain injury (16, 17). In this activated state, microglial cells have increased energy demands, leading to enhanced glucose uptake and metabolism. This metabolic shift is mediated by targets such as lactate dehydrogenase, mammalian target of rapamycin, and hypoxia-inducible factor-1 alpha, which increase glycolysis and lactate release, further exacerbating mitochondrial dysfunction (17). This process may explain the elevated lactate and reduced glucose levels observed in CSF of ABI patients with postoperative infections. However, elevated CSF lactate can also occur without ischemia.

Previous studies indicated that CSF metabolites have high diagnostic accuracy in patients with suspected intracranial infections, particularly bacterial meningitis (18, 19). In this study, mean cGlu and cLac levels significantly differed between infected and non-infected groups. These findings align with studies by Peng et al. (18) and Pesaresi et al. (19). Additionally, the areas under the ROC curve for cGlu and cLac were 0.836 and 0.820, respectively, demonstrating strong predictive capability for postoperative intracranial infection in ABI patients.

Limited studies have explored associations between biomarker concentrations in CSF and blood after ABI surgeries. No significant correlation emerged between bLac and cLac (*r* = 0.071, *P* = 0.502), consistent with previous findings (11). This lack of correlation results from limited lactic acid permeability across the blood-brain and blood-CSF barriers, and the absence of active transport mechanisms for lactate (20). Meanwhile, cGlu was significantly correlated with cLac (*r* = -0.56, *P* < 0.001), suggesting that elevated cLac relates to cerebral hyperglycolysis. However, cLac showed only a weak correlation with CSF PCT (*r* = 0.277, *P* = 0.009), and no significant correlation with cGlu (*r* = -0.100, *P* = 0.352). ROC analysis yielded an AUC of 0.631 for CSF PCT, consistent with previous results (6, 21–23).

Kul et al. (12) examined 56 TBI patients and reported that low CGLR combined with elevated CSF lactate indicated unfavorable outcomes. A retrospective study by Morotti and Goldstein (15), including 114 patients with non-traumatic SAH across five European university intensive care units, identified an independent association between CGLR and poor prognosis. Similarly, a multicenter prospective observational study by Abassi et al. (1), involving 219 ABI patients admitted to 12 European ICUs, concluded that decreased CGLR independently predicted adverse neurological outcomes at 3 months and ICU mortality. Recently, Wang et al. (24) identified CGLR as an early diagnostic biomarker for vasospasm in patients with aneurysmal SAH. Collectively, these studies support the view that CGLR is a versatile biomarker for assessing central nervous system metabolic status, consistent with our finding of strong diagnostic performance of CGLR across different ABI subtypes.

In this study, patients with ABI who developed intracranial infection after brain surgery had significantly lower CGLR values compared to non-infected individuals. The ROC curve analysis yielded an AUC of 0.866 for CGLR, demonstrating superior overall accuracy as a biomarker for intracranial infection compared to cLac or cGlu alone. At an optimal cutoff of 0.59, CGLR achieved a specificity of 94.1% and a sensitivity of 75.6%. Subgroup analyses confirmed consistent diagnostic performance of CGLR in patients with TBI, ICH, and SAH. The optimal cutoff values (0.51–0.61) for these subgroups had extensively overlapping 95% confidence intervals, statistically supporting a unified CGLR cutoff (0.59) for patients with different ABI types. From a clinical perspective, these findings suggest that a CGLR value ≤ 0.59, given its high specificity (94.1%), can help rule in intracranial infection with reasonable confidence, supporting the initiation or continuation of empirical antibiotic therapy. Conversely, a CGLR value > 0.59, with its moderate sensitivity (75.6%), may assist in ruling out infection in patients with low clinical suspicion, potentially allowing deferral or de-escalation of antibiotic therapy while awaiting culture results.

Kul et al. (12) previously reported that a CGLR below 1.47 independently predicted poor prognosis in ABI patients. Abassi et al. (1) identified an optimal CGLR cutoff of 1.39 to predict adverse outcomes at 3 months. The discrepancy in optimal cutoff values between studies may be due to additional metabolic disturbances caused by infection (25, 26), leading to rapid glucose depletion and a significant increase in lactate. Consequently, the cutoff (0.59) for diagnosing infection is lower, suggesting that CGLR sensitively reflects different pathological conditions. To address whether CGLR primarily reflects injury severity rather than infection, we compared CGLR between infected and non-infected patients. CGLR remained significantly lower in infected patients regardless of glycemic status, suggesting an infection-specific component beyond injury severity.

This study confirmed a modest positive correlation between peripheral and CSF glucose (*r* = 0.267, *P* = 0.001), consistent with physiological expectations. However, blood glucose levels did not differ significantly between infected and non-infected groups. Subgroup analysis revealed that CGLR remained significantly lower in infected patients, irrespective of glycemic status (all *P* < 0.001). No significant differences in CGLR appeared between normoglycemic and hyperglycemic patients within the same infection status (*P* = 0.932 for infected; *P* = 0.550 for non-infected). Additionally, a strong inverse correlation between CSF glucose and lactate was observed in infected patients (*r* = -0.549, *P* < 0.001), but not in non-infected individuals (*r* = -0.259, *P* = 0.069), suggesting infection induces a tightly coupled local metabolic state Zhang et al. (27). Soon et al. recently reported excellent diagnostic accuracy of CLGR (AUC 0.994) in neurosurgical patients. However, their cohort had fewer culture-negative infections (8.5%) and excluded many inconclusive cases. In contrast, our cohort included more culture-negative infections (58.5%) and a broader spectrum of ABI etiologies, possibly explaining the relatively lower performance of CLGR in our population (AUC 0.832). In this study, CGLR slightly outperformed CLGR (AUC 0.866 vs. 0.832). A potential explanation is that CGLR, which does not directly depend on peripheral glucose levels, may be less affected by systemic glycemic variations. In contrast, CLGR incorporates the CSF-to-blood glucose ratio and may be more sensitive to stress-induced hyperglycemia or insulin therapy in critically ill ABI patients. Therefore, compared with CLGR, CGLR not only showed higher diagnostic accuracy (AUC 0.866 vs. 0.832) but also eliminates the need for simultaneous blood glucose measurement, reducing clinical inconvenience and potential timing-related errors. Moreover, it is relatively insensitive to peripheral glucose fluctuations and specifically reflects central nervous system infection status, offering distinct advantages over CSF culture (days) or NGS ( > 24 h, high cost).

The current study has several limitations. First, its findings are constrained by a limited sample size and single-center design, requiring multicenter validation to confirm the proposed CGLR cutoff. Second, dynamic analysis of CSF metabolites was not performed, preventing evaluation of the relationship between changes in intracranial infection severity and CSF metabolites. Third, despite controlling for multiple confounding factors, unmeasured variables may remain. Furthermore, since all patients were managed in the ICU due to severe ABI, the findings might not generalize to patients with mild ABI who do not require ICU care. Future studies are needed to validate CGLR in non-ICU settings. Finally, subtle variations in CSF glucose and lactate measurement methods among laboratories may challenge the establishment of a universal cutoff value.

## 5 Conclusion

Although using cLac or cGlu alone demonstrates good diagnostic efficacy for postoperative intracranial infection after ABI, the combined indicator CGLR shows superior diagnostic performance. Additionally, CGLR sensitively reflects various pathological conditions. Moreover, it is relatively insensitive to systemic glycemic fluctuations and more specifically reflects central nervous system infection status. Thus, CGLR may serve as a rapid, practical, and cost-effective biomarker for early diagnosis of postoperative intracranial infections in ABI patients.

## Data Availability

The raw data supporting the conclusions of this article will be made available by the authors, without undue reservation.
